# DaReUS-Loop: accurate loop modeling using fragments from remote or unrelated proteins

**DOI:** 10.1038/s41598-018-32079-w

**Published:** 2018-09-12

**Authors:** Yasaman Karami, Frédéric Guyon, Sjoerd De Vries, Pierre Tufféry

**Affiliations:** Molécules Thérapeutiques in silico, UMR-S973, Institut National de la Santé et de la Recherche Médicale (INSERM), Université Paris Diderot, Sorbonne Paris Cité, RPBS, 75013 Paris, France

## Abstract

Despite efforts during the past decades, loop modeling remains a difficult part of protein structure modeling. Several approaches have been developed in the framework of crystal structures. However, for homology models, the modeling of loops is still far from being solved. We propose DaReUS-Loop, a data-based approach that identifies loop candidates mining the complete set of experimental structures available in the Protein Data Bank. Candidate filtering relies on local conformation profile-profile comparison, together with physico-chemical scoring. Applied to three different template-based test sets, DaReUS-Loop shows significant increase in the number of high-accuracy loops, and significant enhancement for modeling long loops. A special advantage is that our method proposes a prediction confidence score that correlates well with the expected accuracy of the loops. Strikingly, over 50% of successful loop models are derived from unrelated proteins, indicating that fragments under similar constraints tend to adopt similar structure, beyond mere homology.

## Introduction

Prediction of protein structures is one of the challenging problems in biology^1^. This is reflected by the large number of protein sequences known today (about 109 millions) in the Universal Protein Resource (UniProt)^2^ versus the number of known protein structures (about 139 thousands) deposited in Protein Data Bank (PDB)^3^. Such drastic difference is due to the experimental difficulties of X-ray crystallography or NMR, compared to the rapid rate of new sequences being determined by next-generation sequencing methods. Systematic studies of protein classification demonstrated that existing proteins can be grouped into very few homologous families^4–6^. This means homology modeling is a crucial technique to obtain structural insight^7^, and homology modeling methods keep significantly improving^8,9^.

Loops are regions with often crucial roles in protein-protein interactions, protein function, drug design and docking of small molecules^10–12^. On the other hand, in more than one half of deposited structures in PDB missing segments (often loops) are reported^13^, highlighting the importance of loop modeling. Successful loop modeling can lead toward accurate design and engineering of proteins, large peptides, antibodies, drugs or synthetic vaccines, to name a few^14^. Importantly, loop modeling is a crucial step in homology modeling. Loop regions are much more variable in sequence and structure than other regions, leading to larger deviations from the homologous templates^15–19^. Despite the development of dedicated loop modeling methods, the overall accuracy of homology models tends to be considerably lower in loop regions, and loop modeling of homology models remains an open problem^20–23^. Finally, it must be emphasized that loop modeling can encompass different scopes, that range from protein modeling, in which the identification of one native conformation is expected, to the modeling of protein-protein interactions or protein-ligand interactions, in which information about loop conformational variability is desirable^24–29^.

Existing loop modeling methods can be divided into: *ab initio* based^30–35^, knowledge-based^36–38^ and the combination of both methods^39–41^.

*Ab initio* methods determine loop conformations computationally, through the exploration of the conformational space. They are dependent on energy optimization techniques and are consequently highly time consuming. For the completion of crystal structures, Rosetta Next-Generation KIC (NGK)^31^ and GalaxyLoop-PS2^32^ are two state-of-the-art examples of *ab initio* methods that have been shown to provide accurate loop predictions. Rosetta NGK is a robotics-based method using a hybrid energy function with physics-based and knowledge-based energy terms, enabling NGK to find accurate loop candidates. GalaxyLoop-PS2 is also based on a hybrid energy function that concurrently employs the strength of different energy components, considering short-range, hydrophobic and electrostatic interactions.

Data-based methods are dependent on the geometry of flanking residues and the database used for mining candidates^40^. Flanks are regions before and after the loop to be modeled. For the completion of crystal structures, these methods are shown to generate successful results when similar fragments to the loop of interest exist in the database^41^. ArchPRED^42^ considers the secondary structures flanking the missing loop, their relative orientation and the number of missing residues to identify candidate loop conformations. FREAD^43^ searches for candidate fragments matching conditions on distances between C_*α*_ of the flanks. LoopIng^37^ is based on Random Forest model and considers sequence and geometry related features to select the candidates. SuperLooper2^44^ mines the Loop In Protein (LIP) database^45^, a comprehensive loop database containing all protein segments up to 35 residues from the PDB, to identify fragments matching geometrical criteria between the two last atoms of the main chain of one flank and the two first of the other.

Hybrid loop modeling methods combine *ab initio* and data-based methods to improve the quality of loop predictions. CODA generates a consensus loop prediction using both *ab initio* and data-based methods independently^40^. Similar approaches are considered by others to predict complementary determining region (CDR) of antibodies^46,47^. Another recent method is Sphinx, which first performs data-based search to find fragments shorter than the loop of interest and obtains structural informations^41^. Then it applies *ab initio* methods to generate fragments of correct length.

Most of the existing loop modeling methods are shown to perform successful loop predictions in high-resolution crystal structures with accuracies of about 1-2*Å*, if the loop is short (3–12 residues)^32–34,37,41,43,44^ and increasing up to ~4*Å* for larger sizes (≤20 amino acids)^37,41,43,44^. However, in practical applications, loops of interest are typically non-homologous regions of a homologous template. For instance, data-based methods perform the search considering flank residues. In high-resolution crystal structures, these flanks are perfect. In contrast, flanks derived from homologous templates might represent very large root-mean-square deviations (RMSD) to the native flanks. Very few studies have tackled method assessment in such perturbed situations and their accuracies are about 1–4*Å* for short loops (3–12 residues)^32,37,43^ but decrease significantly (4–9*Å*) for larger sizes (13–15 amino acids)^43^.

Another challenging, yet unsolved problem is the prediction of long loops: many of existing loop modeling methods have been designed to predict loops of at most 12 residues.

We previously introduced a fast and efficient approach to mine large collections of structures using a Binet-Cauchy kernel, to search for similar fragments without gaps^48^. It was extended to the search for loop candidate given loop flanks, BCLoopSearch^49^. However, according to our early tests, the following bottlenecks need to be tackled. First, to propose a strategy to prune the possibly very large number of candidates. Next, despite the fact that Binet-Cauchy kernel can tolerate some distortion, a sub-optimal geometry of the flanks can lead to failures in returning the right loop conformation. Finally, the accurate scoring of the loops is still an issue.

In this study we propose DaReUS-Loop (Data-based approach using Remote or Unrelated Structures for Loop modeling). DaReUS-Loop tackles the practical application of loop modeling in non-ideal conditions. Considering the flanks, we mine the entire set of protein entries in the PDB and extract similar fragments. Then we prune the set of candidates considering their sequence similarity and conformational profile. Finally, we build complete protein models and rank them. Our scoring schema provides us with a final set of 10 best models.

We evaluated our method on three challenging template-based test sets: CASP11, CASP12 and HOMSTRAD. The large number of results with RMSD less than 2*Å* suggests the accuracy of our method predicting loops in a homology modeling context. To assess the quality of the results, we compared our approach with two state-of-the-art *ab initio* methods, Rosetta NGK and GalaxyLoop-PS2, one data-based method, LoopIng and Sphinx, that is a hybrid method. Comparisons represent that our protocol performs equally or better than those other methods. In addition, DaReUS-Loop outperforms the other approaches to predict long loops of at least 15 residues. A special advantage is that our method proposes a prediction confidence index that correlates well with the expected accuracy of the loops. The computing time of our method is substantially less than Rosetta NGK, GalaxyLoop-PS2 and Sphinx. Strikingly, almost all successful loop models are derived from unrelated proteins, indicating that fragments under similar constraints tend to adopt similar structure, beyond mere homology.

## Results

**Figure 1:**
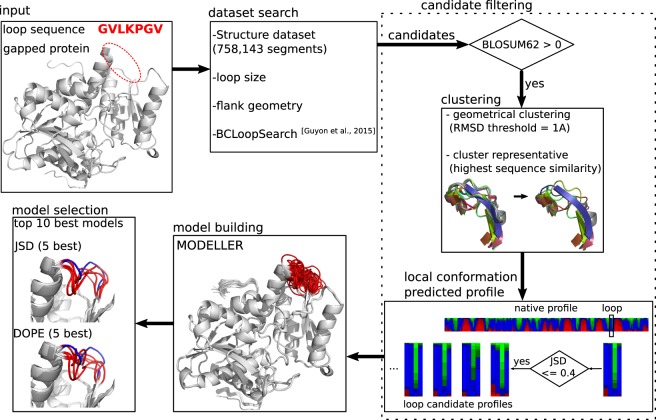
DaReUS-Loop workflow. The workflow describes main steps of the loop modeling protocol: loop candidate search, candidate filtering, model building and model selection. The inputs are a gapped structure and loop sequence. In the final step, two measures are considered for scoring the models. The 5 best models scored by each measured are returned as the final predictions.

Figure 1 summarizes the workflow of our approach. Given the input of a gapped structure (PDB format) and the complete sequence to model, a first step is to identify loop candidates from the loop flanks using BCLoopSearch, mining a set of PDB structures. Due to the possibly very large number of candidates, clustering and filtering are applied to reduce the number of candidates. Three types of filters involve loop sequence similarity, local geometry and conformational profile comparison. Finally, models are built and the 10 best scored models are returned.

### Effects of the filtering

**Figure 2:**
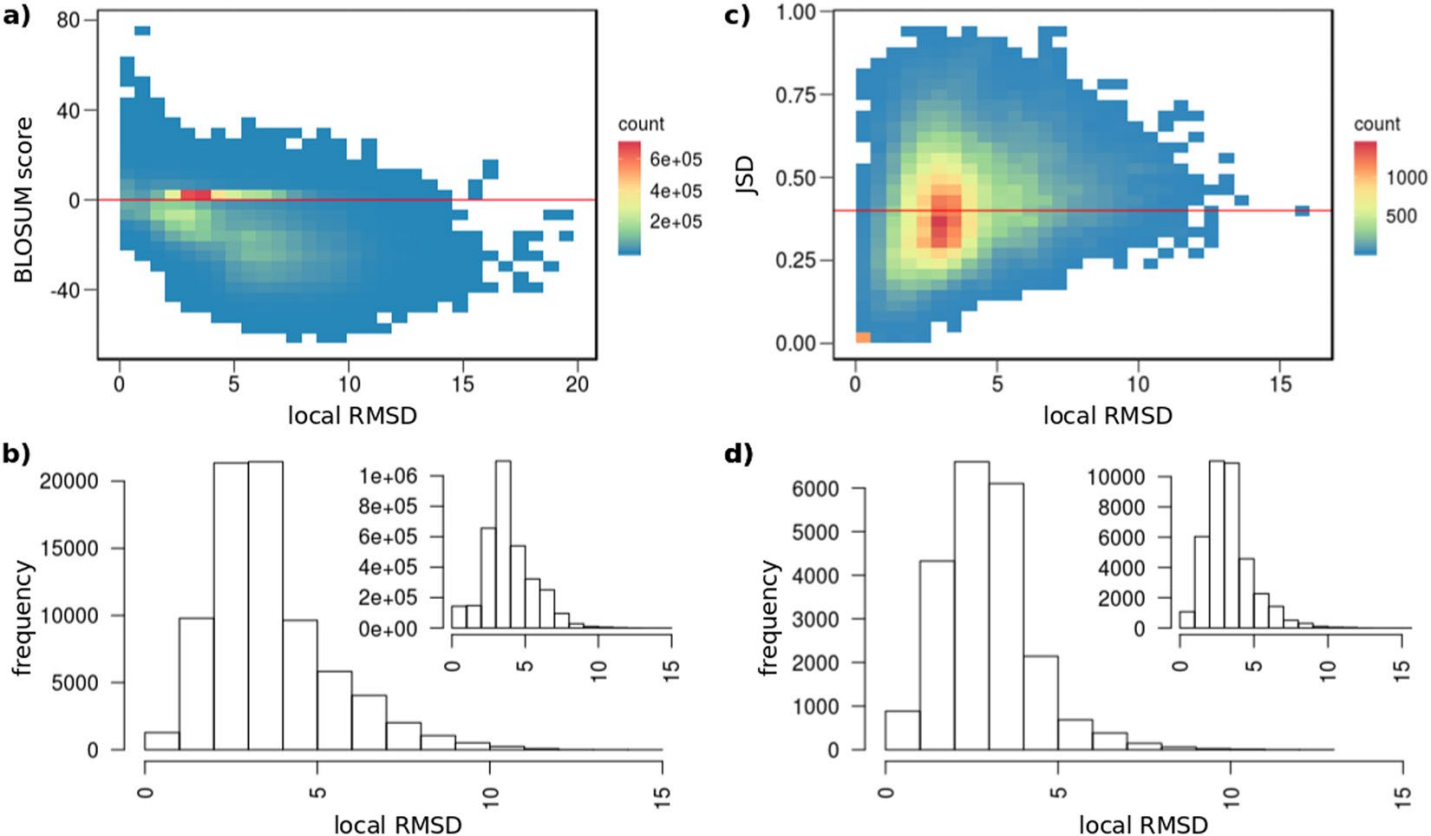
Analyzing the effect of filtering for CASP11 test sets. Four different filtering methods were sequentially applied in our protocol. We report the result of each filter for the loops of CASP11 test sets: (**a**) sequence similarity, (**b**) geometrical clustering, (**c**) predicted local conformation, (**d**) structural clashes. The smaller histograms on the top right of **b** and **d**, represent the local RMSDs before applying the corresponding filters.

In this section we report the effect of filtering over the set of all loop candidates retrieved from our dataset for CASP11 test set. The distribution of sequence identity (BLOSUM scores) with respect to loop local RMSD are shown in Fig. 2a. 36% of the candidates have positive BLOSUM scores and 62% of them have local RMSDs of less than 4*Å*. In total, this step makes the fraction of fragments with RMSDs less than 4*Å* increase from 49% before filtering up to 62%. Figure 2b depicts the impact of clustering. As expected, it results in a drastic decrease of the number of candidates. It also comes with a slight improvement in terms of the RMSDs. The mean (resp. median) RMSD is of 3.86 (resp. 3.60)*Å* before clustering and of 3.60 (resp. 3.24)*Å* after. As an outcome, 70% of the candidates selected have a RMSD value <4*Å*. Figure 2c represents the distribution of remaining loops local RMSD values with respect to their Jensen Shannon Divergence (*JSD*) values. At this stage, 52% of the candidates have *JSD* >0.40 and 65% of candidates with high local RMSD (>4*Å*), have also high *JSD* (>0.40). Filtering out candidates with JSD values more than 0.40 results in improving the fraction of candidates with a RMSD less than 4*Å* from 70% up to 74%. Finally, the last filter consists of discarding candidates that have clashes after modeling Fig. 2d. This improves the average local RMSDs from 3.29*Å* to 2.94*Å*. After all filters have been applied, 84% of the final set of candidates have local RMSD <4*Å*.

### Quality of the predictions

We compared DaReUS-Loop to two state-of-the-art *ab initio* methods, Rosetta NGK and GalaxyLoop-PS2, one data-based method, LoopIng and a hybrid method, Sphinx on the common sub-set of loops that could be predicted by all the methods (Common_*ai*_ and Common_*db*_, respectively). Overall statistics on the best of top 10 models are shown in Table 1; more detailed results including per-model local and global RMSDs are reported in Table 2. On average, the DaReUS-Loop protocol outperforms Rosetta NGK and GalaxyLoop-PS2 by at least 0.25, 0.36, and 0.33*Å*, for the CASP11, CASP12, and HOMSTRAD benchmark sets, respectively. Apart from HOMSTRAD, one also notes that the RMSDs are rather close to the best possible values for the CASP11 and CASP12 sets, with a loss of only 0.40 and 0.56*Å*, respectively. A larger deviation of 0.73*Å* is observed for the HOMSTRAD set. Looking at the comparisons with data-based methods (Common_*db*_ set) for DaReUS-Loop, one observes an increase of the flanked RMSD values, i.e. 0.21, 0.34, and 0.15*Å* for CASP11, CASP12, and HOMSTRAD, respectively compared to the values obtained for the Common_*ai*_ subset. This results from reducing flank size to only 2 amino acids per loop end, instead of 4. Moreover, DaReUS-Loop outperforms LoopIng for all sets, with a gain of at least 1*Å* in all cases. Finally, DaReUS-Loop outperforms Sphinx by at least 0.70*Å* for the CASP11 and CASP12 test sets, while only a slight improvement is observed for the HOMSTRAD test set. In addition, we report the average flanked RMSD values, while selecting the top 10 models using either *JSD* or *DOPE* in Table 2. We observed that both scores result in rather similar predictions, however considering the two together, brings improvements.

**Table 1:** Prediction results over the top 10 models.

Subset	Method	CASP11	CASP12	HOMSTRAD	<*Å* (%)	<2*Å* (%)
Common_*ai*_	best	2.18	2.31	1.65	24	66
	DaReUS-Loop	**2.58**	**2.87**	**2.38**	**19**	**47**
	Rosetta NGK	2.96	3.34	2.71	12	35
	GalaxyLoop-PS2	2.83	3.23	2.96	13	36
Common_*db*_	DaReUS-Loop	**2.79**	**3.21**	**2.53**	**14**	**44**
	LoopIng	4.35	4.20	4.50	8	16
	Sphinx	3.71	3.94	2.63	12	37
CommonHC_*ai*_	best	1.43	1.63	1.65	28	76
	DaReUS-Loop	**1.91**	**2.30**	**2.38**	**22**	**54**
	Rosetta NGK	2.59	2.99	2.71	14	38
	GalaxyLoop-PS2	2.34	2.88	2.96	15	41
CommonHC_*db*_	DaReUS-Loop	**2.05**	**2.25**	**2.53**	**17**	**53**
	LoopIng	3.66	3.53	4.50	10	20
	Sphinx	2.90	3.19	2.63	14	42

Average flanked RMSD (*Å*) are reported for the CASP11, CASP12 and HOMSTRAD test sets, over the Common and CommonHC subsets. Comparison is between DaReUS-Loop, *ab initio* (_*ai*_) methods (Rosetta NGK and GalaxyLoop-PS2) and data-based (_*db*_) methods (LoopIng and Sphinx). All the RMSD values reported in this table correspond to the best flanked RMSD (*Å*) over 10 models. The “best” row shows the best candidate loop identified by DaReUS-Loop, before applying the filters or the top 10 selection. For Common_*ai*_ and CommonHC_*ai*_ (resp. Common_*db*_ and CommonHC_*db*_), the flanked RMSDs are calculated using flanks of 4 (reps. 2) amino acids. The percentage of highly accurate predictions (<1*Å* and <2*Å*) is also reported. Bold values correspond to the best values among all the methods.

**Table 2:** Detailed comparison of the results.

Test-set	Subset	Method	Local RMSD (*Å*)			Flanked RMSD (*Å*)			Global RMSD (*Å*)		
			Average	Std	Median	Average	Std	Median	Average	Std	Median
CASP11	CommonHC_*ai*_	best	0.78	0.38	0.74	1.47	0.82	1.30	4.11	3.53	2.52
		JSD	1.05	0.59	0.89	1.97	1.37	1.57	4.74	3.99	3.10
		DOPE	1.19	0.59	1.09	2.19	1.14	2.04	4.94	3.97	3.31
		DaReUS-Loop	**1.00**	0.53	0.89	**1.91**	**1.33**	**1.66**	**4.71**	**4.08**	**2.77**
		Rosetta-NGk	1.44	0.82	1.22	2.59	1.40	2.47	5.33	4.37	3.5
		GalaxyLoop-PS2	1.34	0.70	1.16	2.34	1.32	2.54	5.34	4.32	3.07
	CommonHC_*db*_	best	0.92	0.66	0.78	1.56	1.15	1.19	3.91	3.26	2.49
		JSD	1.23	0.94	0.96	2.11	1.58	1.59	4.57	3.69	2.8
		DOPE	1.29	0.80	1.09	2.23	1.46	1.95	4.74	3.70	3.00
		DaReUS-Loop	**1.19**	0.91	0.96	**2.05**	1.54	1.59	**4.54**	3.78	2.75
		LoopIng	1.94	1.12	1.84	3.66	2.08	3.35	—	—	—
		Sphinx	1.47	1.00	1.24	2.90	2.15	2.40	5.40	4.17	4.14
CASP12	CommonHC_*ai*_	best	0.90	0.60	0.75	1.63	0.99	1.38	3.07	2.51	2.24
		JSD	1.27	0.93	1.04	2.47	1.98	1.82	3.92	2.95	2.84
		DOPE	1.25	0.74	1.20	2.46	1.64	2.00	3.84	2.76	2.90
		DaReUS-Loop	**1.21**	**0.86**	**0.97**	**2.30**	**1.63**	**1.87**	**3.81**	**2.78**	**3.21**
		Rosetta-NGk	1.53	0.98	1.49	2.99	2.88	2.33	4.20	3.81	3.37
		GalaxyLoop-PS2	1.43	0.99	1.15	2.88	2.88	1.98	4.34	3.86	3.27
	CommonHC_*db*_	best	0.89	0.57	0.75	3.87	3.96	2.36	1.57	0.92	1.33
		JSD	1.28	0.87	1.06	2.39	1.81	1.83	4.85	4.71	3.18
		DOPE	1.22	0.70	1.17	2.36	1.44	2.03	4.77	4.57	3.12
		DaReUS-Loop	**1.22**	0.80	0.99	**2.25**	1.54	1.84	**4.74**	4.53	3.63
		LoopIng	1.72	1.06	1.61	3.53	2.24	3.28	—	—	—
		Sphinx	1.47	0.85	1.32	3.19	3.00	2.39	5.25	5.25	3.57
HOMSTRAD	CommonHC_*ai*_	best	0.93	0.48	0.87	1.65	0.68	1.56	2.24	0.69	2.17
		JSD	1.25	0.57	1.14	2.34	1.27	2.16	3.09	1.42	2.94
		DOPE	1.23	0.58	1.01	2.12	0.93	2.18	2.68	0.84	2.69
		DaReUS-Loop	**1.26**	**0.59**	**1.16**	**2.38**	**1.12**	**2.55**	**2.88**	**1.00**	**2.92**
		Rosetta-NGk	1.58	0.65	1.69	2.71	1.36	2.66	3.40	1.51	3.10
		GalaxyLoop-PS2	1.34	0.61	1.34	2.96	1.70	2.66	3.68	1.92	2.87
	CommonHC_*db*_	best	0.95	0.50	0.87	1.72	0.76	1.61	2.28	0.76	2.16
		JSD	1.44	0.68	1.35	2.72	1.44	2.50	3.37	1.53	3.04
		DOPE	1.36	0.68	1.19	2.34	1.10	2.16	2.81	0.96	2.79
		DaReUS-Loop	**1.45**	0.71	1.45	**2.53**	1.16	2.57	**2.98**	1.09	2.93
		LoopIng	2.16	0.58	2.23	4.50	1.74	4.16	—	—	—
		Sphinx	1.51	0.94	1.45	2.63	1.65	2.29	3.53	1.71	2.86

The average and its standard deviation and median RMSD values (10 models, *Å*) are reported. The RMSDs are calculated as root-mean-square deviation of the candidate loop main-chain atoms N, C_*α*_, C and O to the native loop. Bold values correspond to the best average values among all the methods.

Considering the performance using only the top models, since DaReUS-Loop is based on both JSD and DOPE, we selected for each loop the top sccoring models by DOPE and the top model scored by JSD, and chose the best out of the two. To keep the comparison fair, we compared our results with the best of top 2 predicted by Rosetta-NGK and Sphinx and results are reported in Supplementary Table S1 - the other methods (GalaxyLoop-PS2 and LoopIng) do not provide the scores of the models. The results show that DaReUS-Loop performs better than Rosetta-NGK and Sphinx in almost all the cases, the only exception being for the HOMSTRAD test set, where Sphinx performs slightly better than DaReUS-Loop - note that the loops of the HOMSTRAD set are, on average shorter than those of the CASP11 and CASP12 sets.

### Prediction confidence index

**Figure 3:**
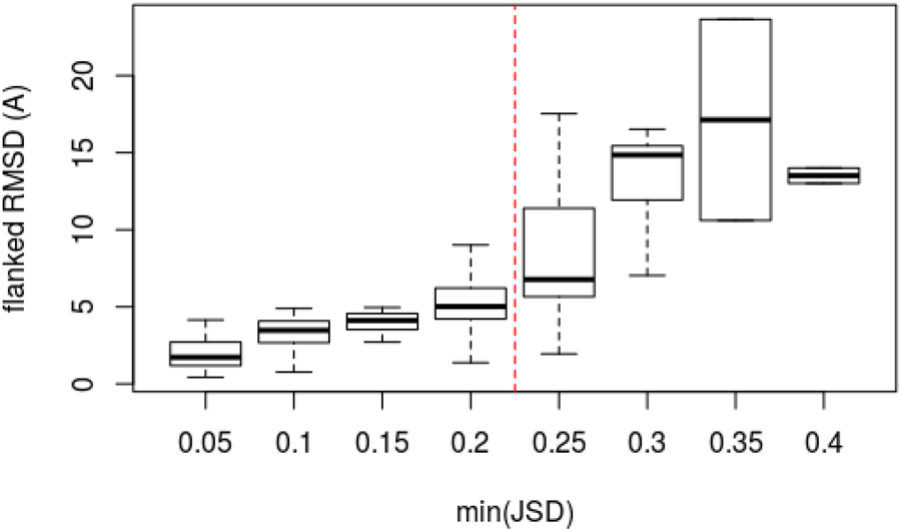
The correlation between *min*(*JSD*) and flanked RMSD. For the sake of clarity, *min*(*JSD*) values are depicted as bins with a width of 0.05. The flanked RMSD is for the best DaReUS-Loop candidate out of top 10 models. The correlation is shown for 142 loops of the CASP test sets. Boxes span the interquartile range (IQR) from 25th to 75th percentile and the thick black lines represent median values (50th percentile). The whiskers extend to furthest values within 1.5 times the IQR from the box.

We now turn to analyzing whether a prediction confidence could be assigned based on the *min*(*JSD*) score, which indicates the best fit of any candidate loop in terms of conformational profile. Figure 3 shows a clear trend that lower *min*(*JSD*) values are associated with lower RMSDs, with a Spearman correlation of 0.76. From the figure one also observes a clear jump in the range of RMSD values between *min*(*JSD*) of 0.20 and 0.25, and for JSD values more than 0.20, the quality of the correlation appears degraded. This analysis suggests that *min*(*JSD*) can be considered as a measure to assess the overall case-by-case loop modeling quality and to detect failures of our protocol. Therefore, for each of the three datasets, a high-confidence subset was selected (CommonHC), discarding any loop target for which the *min*(*JSD*) is more than 0.20 (14 loops in CASP11 and 16 loops in CASP12 test sets) Table 1. For the HOMSTRAD set, all loops of the Common subset meet the condition of a JSD less than 0.20, and the results are unchanged. For the CASP11 and CASP12 sets, one clearly sees a decrease of the average RMSDs by more than 0.55*Å*, and the values appear closer to that obtained for HOMSTRAD. The performance of DaReUS-Loop compared to other methods (Rosetta NGK, GalaxyLoop-PS2, LoopIng and Sphinx) remains almost unaffected.

### Modeling loops at high accuracy

**Figure 4:**
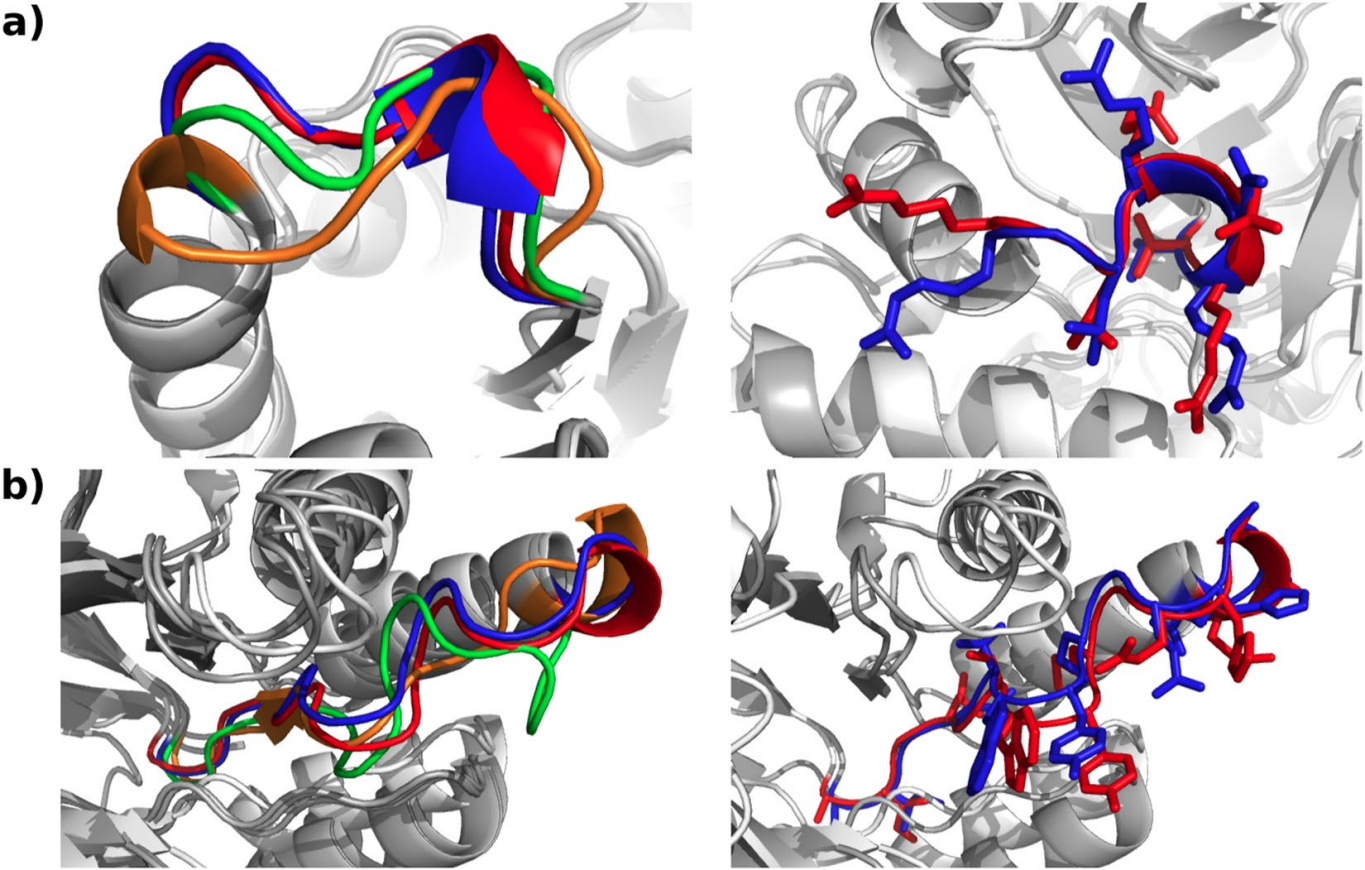
Examples of the predictions. The results of DaReUS-Loop (blue), Rosetta NGK (green), GalaxyLoop-PS2 (orange) and crystal structure (red) are illustrated for two loops. The two loops belong to target T0807 of CASP11, (**a**) a loop of length 7 and (**b**) length 15. The RMSD of each predicted loop compared to the native loop is reported as (**a**) DaReUS-Loop: 0.9*Å*, NGK: 1.5*Å* and PS2: 2.9*Å* and (**b**) DaReUS-Loop: 1.3*Å*, NGK: 3*Å*, PS2: 2.9*Å*. On the right column the side chains of the native and predicted loops by DaReUS-Loop are shown.

DaReUS-Loop generates high-accuracy loop models (<1*Å*) for 23 (19%) and medium-accuracy models (<2*Å*) for 57 (47%) of the cases in the Common_*ai*_ subset (Table 1). This success rate is very satisfactory considering the fact that before filtering, for only 29 loops (24%) a high-accuracy candidate is found in the fragment database, limiting the maximum success rate. For medium-accuracy models, the maximum success rate is 80 cases (66%). The results for high and medium accuracy constitute an improvement by 7 and 12% over Rosetta NGK and 6 and 11% over GalaxyLoop-PS2. For the Common_*db*_ subset, the improvements are of 6% (9/153) and 28% (43/153), respectively, over LoopIng and 2% (4/153) and 7% (12/153) over Sphinx. Illustrative examples are shown in Fig. 4 and Supplementary Figure S1. For DaReUS-Loop and the other methods, the CommonHC subset retains essentially all of the high-accuracy and medium-accuracy loop models. For DaReUS-Loop, this increases the success rate to 22% and 54% for high-accuracy and medium-accuracy loops, respectively.

### Modeling long loops

**Figure 5:**
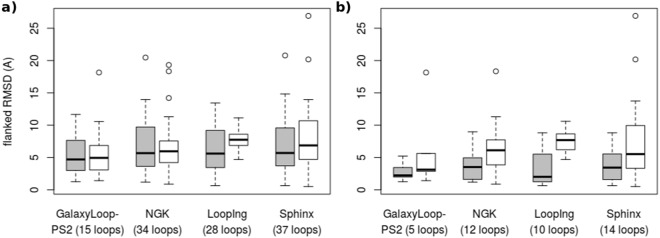
Flanked RMSD (*Å*) of long loops in CASP11 and CASP12 test sets. The results are compared with GalaxyLoop-PS2, Rosetta NGK, LoopIng and Sphinx. a (resp. b): results obtained for long loops of the Common (resp. CommonHC) subset. DaReUS-Loop results are colored in gray and the other methods are shown in white. Boxes span the interquartile range (IQR) from 25th to 75th percentile and the thick black lines represent median values (50th percentile). The whiskers extend to furthest values within 1.5 times the IQR from the box and circles are outliers.

We now analyze more in details the results obtained for long loops, a challenging and unsolved problem. To assess it, we consider loops with a size of at least 15 residues. Results are presented in Fig. 5, and detailed results for each method are reported in Supplementary Table S2. Since the number of such loops common to all methods is very low, to maximize the size of the sample, we present independent pairwise comparisons of DaReUS-Loop with NGK, Galaxy, LoopIng and Sphinx. For the Common subset, DaReUs-Loop outperforms LoopIng and Sphinx, the two methods relying on a databank search, with average improvements of 1.83 and 1.5*Å*, respectively. It performs slightly better than NGK and Galaxy with improvements of 0.21 and 0.47*Å*, respectively. One observes some outliers among the predictions of NGK, GalaxyLoop-PS2, Sphinx and DaReUS-Loop. Indeed, DaReUS-Loop can model almost all the long loops in the test sets and its failure rate is 3% (1/37) compared to 6% (2/37) for Sphinx, 7% (1/15) for GalaxyLoop-PS2 and 9% (3/34) for NGK. Excluding those cases, the performance of DaReUS-Loop remains better than Sphinx by 0.81*Å* n while, NGK and GalaxyLoop-PS2 perform better by 0.11 and 0.63*Å*. Note that, this is an average performance and in some cases, DaReUS-Loop is able to provide solutions when NGK and GalaxyLoop-PS2 fail. For the CommonHC subset, on the other hand, DaReUS-Loop performs significantly better than GalaxyLoop-PS2, Rosetta NGK, LoopIng and Sphinx by 3.01, 3.41, 4.32*Å* and 3.98*Å*, respectively. In the absence of the outliers (none for DaReUS-Loop and LoopIng) the performance of DaReUS-Loop remains better than Rosetta NGK, GalaxyLoop-PS2 and Sphinx by 1.82, 0.28 and 1.42*Å*, respectively. Finally, we conclude that for high-confidence targets, the overall accuracy of DaReUS-Loop to model long loops is notably better.

### Loop candidates are selected from remote or unrelated proteins

**Figure 6:**
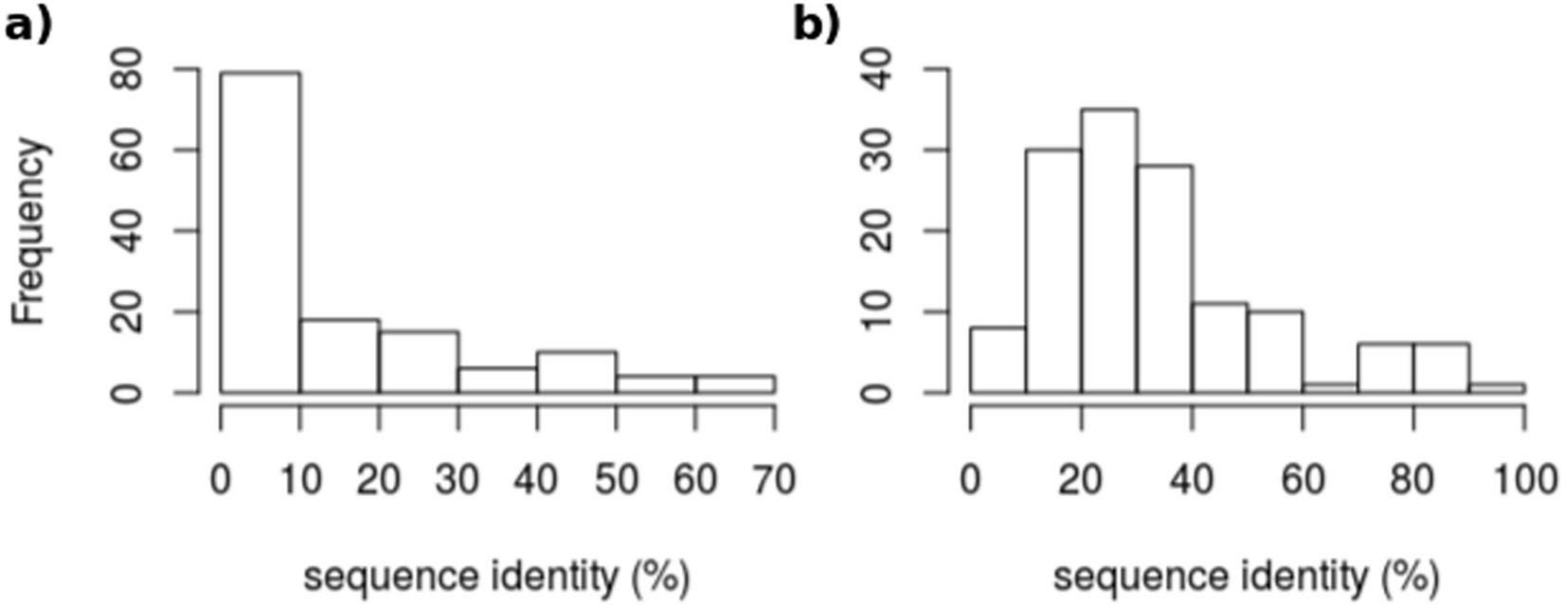
The frequency of sequence identity for all the loops in the test sets (135 loops). The distribution of sequence identity between the best over top 10 loop candidate and target proteins is shown (**a**) over the complete sequence and (**b**) over the loop regions only.

Figure 6 shows the distribution of the sequence identity between the proteins in which the candidates are selected and the target proteins. For 58% (79 out of 135) of the cases, loop candidates come from proteins with a sequence identity of at most 10%. Considering a sequence identity of at most 20%, this number increases up to 71% (97/135). Only 6% (8/135) of the loop candidates are selected from protein chains with more than 50% sequence identity. We have also analyzed homology in terms of Class Architecture Topology Homology (CATH) classification^5^ (http://www.biochem.ucl.ac.uk/bsm/cath/). We observe that 49% (66/135) of the loop candidates come from protein chains that have not been assigned to a CATH class. We report the results over the remaining 51% (69/135). For 42% (29/69) of the cases, loop candidates were retrieved from other classes, 54% (37/69) from different architecture, 56% (39/69) different topologies and 59% (41/69) were retrieved from different homologous superfamilies. This clearly shows that a large majority of loop hits are chosen from dissimilar or very distant proteins. The loop themselves however have a higher sequence identity, which is not surprising given our filtering procedure.

## Discussion

Here, we propose DaReUS-Loop, a data-based approach that identifies loop candidates from remote or unrelated proteins. DaReUS-Loop is able to mine the complete PDB, employing filters based on sequence similarity, clustering, conformational profiles (based on a structural alphabet) and local geometry to narrow down the candidates. A combination of conformational profiles and atomic-distance-dependent potential (DOPE) is then used to select the best candidates. DaReUS-Loop is specifically designed for loop modeling of structures modeled from homologous templates, when no crystal structure is available. We tested DaReUS-Loop on three challenging template-based test sets and compared the results with the state-of-the-art *ab initio* and data-based loop modeling methods. We also verified that the loops in our benchmarks correspond to surface-exposed loops (see Methods). Results suggest that DaReUS-Loop improves the accuracy of template-based loop prediction by 0.5*Å* on average. Specifically, our method showed a considerable increase in the number of high-accuracy (<1*Å*) loops. This increase in the precision of template-based loop modeling has high importance, specially in the field of drug design. To assess the significance of the improvement, we have used a Wilcoxon signed-rank test^50^ over the flanked RMSD values. With the exception of GalaxyLoop-PS2 in the Common_*ai*_ sub set (*p-value* = 0.17325), the evaluations suggest significant differences between DaReUS-Loop and all the other methods (Rosetta NGK, GalaxyLoop-PS2, LoopIng and Sphinx) in both common and high confidence common sub-sets with 0%≤ *p-value* < 2%.

In addition, DaReUS-Loop is relatively fast with respect to other loop modeling methods. The protocol can take 10–40 minutes (using 40 threads of a 2.2-GHz Intel Xeon processor). The CPU-time needed for DaReUS-Loop is in the range of 10 min to 25 hours, (CPU-time: BCLoopSearch 1–10 min, clustering 1–5 min, local conformation 3 min, local conformation filtering 15 s per candidate and MODELLER 30–50 s per candidate). It has to be stated that in rare cases the number of possible loop candidates might be very large (several hundreds of thousands), consequently this leads to proportional increase in the computational time. Such increase is mostly due to the computations of MODELLER. It has to be mentioned that we pre-computed the local conformation profiles for all the protein chains in our structure dataset, otherwise the computational cost of this step is 3 minutes for every candidate. LoopIng webserver is very fast and modeling a loop costs on average 1 minute. Whereas several days are needed for Rosetta NGK to generate 500 models, depending on the size of the loop and protein (CPU-time: 120–1200 hours). The computational time of GalaxyLoop-PS2 varies between 1 to 4 hours (CPU-time: 8–32 hours) to generate 5 candidates using GalaxyWEB, depending on the size of loop and protein. The performance of Sphinx web-server depends on the length of the loop to be modeled and varies between 20 minutes up to several hours for long loops.

Until now, very few studies have considered loop modeling of template-based models, which highlights the difficulty of the task. While assessing Looping, the authors reported very little performance differences between modeling native and template-based loops of CASP10^37^, which might be explained by *(i)* the short length of the studied loops (between 4 and 8 residues), *(ii)* quality of the models *and (iii)* considering the best results for the evaluations. Park *et al*. evaluated their method (GalaxyLoop-PS2) in different environmental conditions (crystal structure, side-chain perturbed, backbone perturbed and template-based models) and results demonstrated far less accuracy in the case of large environmental errors^32^. Rather similar observations are reported in^43^ to compare the results of loop modeling on CASP 7 and 8, using template-based models versus crystal structures.

A special advantage is that DaReUS-Loop comes with a prediction confidence score that correlates well with the expected accuracy of the loops. This score, based on the best fit in terms of conformational profile, enables us to decide if the modeling procedure was successful or not, bringing some insight about the quality of the final model. In particular, all high-quality and medium-quality loops modeled by DaReUS-Loop belonged to the high-confidence subset. Moreover, for the high-confidence subset, long loops (≥15 residues) modeled by DaReUS-Loop tend to be more accurate compared to other methods. Modeling long loops has been an unsolved problem, most existing approaches dealing with loops of at most 12 residues. Our protocol tackles this problem and improves the accuracy of modeling long loops, as long as high-confidence loop candidates are available from the database.

For the CASP test sets, we extended the gaps to regions between two secondary structures. Such extension can bring two negative consequences: *(i)* the loop gets longer (and therefore harder) and *(ii)* it decreases the chances to find a high-confidence loop candidate. However, the results showed that DaReUS-Loop models long loops with higher accuracy compared to the other methods. On the other hand, we were able to find high-confidence loop candidates in 82% (135/165) of the cases.

Another striking result is that almost all successful loop models are derived from proteins where the homology is remote at best, with low sequence identities and considerable differences in structural classification. In fact, most successful loop models are derived from completely unrelated proteins, with no detectable homology in sequence or structure. The loops themselves have a higher sequence identity, which is expected given our filtering procedure. However, even so, the sequence identities remain quite low, and it is the constraints imposed by the conformational profile (based on the structural alphabet) and by the chemical environment (as measured by the DOPE score) that are the driving force for the selection of the final models. Thus, our results indicate that fragments under similar constraints tend to adopt similar structure, even in the absence of any detectable homology.

## Methods

### Structure Database

Our database to search for loop candidates consists of the entire set of protein structures available in the Protein Data Bank (PDB). In March 2017, it consisted of 123,417 PDB entries, corresponding to 338,613 chains in total. Each chain was split into segments that correspond to consecutive regions separated by gaps or non-standard residues, but accepting seleno-methionines. This led to a database of 758,143 protein segments.

### Template-based test sets

To assess the performance of our approach, we have used three test sets. The first one (HOMSTRAD) was taken from the study by^32^. It consists of 23 loops with sizes between 6 and 11 residues. The two other ones correspond to the targets of the CASP11 (http://predictioncenter.org/casp11/) and CASP12 (http://predictioncenter.org/casp12/) experiments^51,52^. For each CASP target, templates were identified using *HHsearch*^53^ against the *PDB70* database (02-04-2016), considering a maximum sequence identity cutoff of 50% between template and target. In case of multiple, non-overlapping templates, they were combined into a template set. For each target, the template set was aligned to the target using *TM-align*^54^, and the template set with the highest *TM-score* was selected. Only targets where this template set had a *TM* − *score* > 0.5 were retained. This resulted in 12 targets of CASP 11 (out of 46 targets) and 10 targets of CASP 12 (out of 34 targets). For each target, one model was built by MODELLER^7^ using the best template set, with the alignment from *TM-align*. Then, loops were identified as regions of 5 to 30 residues connecting secondary structures of at least 4 residues, as defined by DSSP^55^. Loops that correspond to chain breaks in the experimental structure were excluded. This resulted in a collection of 69 loops and 76 loops for the CASP11 and the CASP12 set, respectively.

The average RMSD of the flanks of the template structure compared to that of the experimental structure of the target is of 0.97*Å*, 1.04*Å* and 0.93*Å* for the CASP11, CASP12 and HOMSTRAD sets, respectively. Loop sizes are between 5–29, 5–28 and 5–11 amino acids for the CASP11, CASP12 and HOMSTRAD test sets, respectively.

### Loop candidate search

We previously introduced the BCLoopSearch protocol, to mine large protein structure datasets and retrieve loop candidates, given two disjoint fragments (loop flanks)^49^. It is based on a Binet-Cauchy (BC) kernel and a Rigidity score:

1 $$BC(X,Y)=\frac{det({X}^{T}Y)}{\sqrt{det({X}^{T}X)det({Y}^{T}Y)}}$$

where *X* and *Y* are C_*α*_ coordinates of the flanks and dataset fragments, respectively and they are centered at the origin. Note that a BC score of 1 indicates a perfect match. *Rigidity* score *R*(*X*, *Y*) is defined as:

2 $$R^{\prime} (X,Y)=ma{x}_{1\le i\le N}|\parallel {X}_{i}-{Y}_{i}\parallel |$$

3 $$R(X,Y)=max\{R^{\prime} (X,Y),|\parallel {X}_{N}-{X}_{1}\parallel -\parallel {Y}_{N}-{Y}_{1}\parallel |\}$$

where *X*_*i*_ and *Y*_*i*_ are C_*α*_ coordinates of the *i*th residues of the flanks and dataset fragments and $||\,\cdot \,||$ is the euclidean norm. Rigidity score is the maximum variation of intra-distances between: *(i)* residues and geometric center and *(ii)* intra-distances between terminal C_*α*_. In addition, we also measured the RMSD between query and candidate flanks for the fragments returned.

In total, four cut-offs values related to *(i)* flank size, *(ii)* flank BC score, *(iii)* flank Rigidity and *(iv)* flank RMSD, have been considered to limit the number of loop candidates. In this study we used: a flank size of 4 residues, Rigidity ≤ 3 and flank RMSD ≤ 4*Å*. The minimal flank BC score cut-off was set depending on the size of the loop to be modeled: 0.9 for loops of at most 8 residues and 0.8 for longer loops.

For each target protein, prior to the loop modeling homologous proteins with more than 70% chain sequence identity were excluded from our search database.

### Candidate filtering

In most cases the number of candidates returned by BCLoopSearch is too large to be tractable, which implies to limit their number. Three filters were sequentially applied in our protocol to this aim:

#### Sequence similarity

The sequence similarity of a loop candidate with the query loop sequence using BLOSUM62 score. Candidates with negative scores were discarded.

#### Geometrical clustering

We used the python Numpy library to measure the pairwise distances (RMSD) between all the candidates^56^. In addition, we used the python Scipy package to perform hierarchical clustering^57^. A RMSD cut-off of 1*Å* was used to group similar loop candidates. To consider memory constraints, we applied an iterative clustering over subsets of 25,000 candidates, until at most 25,000 clusters were obtained. Finally, one representative loop candidate with the highest sequence similarity to the query loop was selected for each cluster. The computational time of our clustering protocol is in the range of 1–5 minutes, however it depends directly on the number of candidates detected by BCLoopSearch. In extreme cases, the needed time may increase up to 10–15 minutes.

#### Local conformation

Previously, Shen *et al*. have shown that local conformation profiles predicted from sequence and profile-profile comparison can be employed to accurately distinguish similar structural fragments^58^. Consequently, we pre-computed a collection of profiles for all the protein chains in the structure dataset, and for all proteins of the test sets. For each loop candidate, it is thus possible to extract the sub-profiles *P* and *Q*, corresponding to the query and candidate loop, and to measure the Jensen Shannon divergence (*JS*(*P*, *Q*)) between these profiles:

4 $$JS(P,Q)=\frac{1}{2}{D}_{KL}(P,M)+\frac{1}{2}{D}_{KL}(Q,M)$$

where *M* corresponds to 1/2(*P* + *Q*) and *D*_*KL*_ is the Kullback-Leibler divergence:

5 $${D}_{KL}(P,Q)=\sum _{1\le i\le 27}P(i)ln(P(i)/Q(i))$$

*P*(*i*) is the probability of SA letter *i*. Then we measured the average Jensen Shannon divergence (*JSD*) over the paired series of query and candidate profiles:

6 $$JSD(P,Q)=\sum _{1\le i\le n}JS({P}_{i},{Q}_{i})/n$$

where *P*_*i*_ and *Q*_*j*_ are the two profiles corresponding to positions 1 to L on the query and candidate loop sequences. Note that a *JSD* of 0 indicates a perfect identity of the profiles. This procedure was applied on each loop candidate and those with a *JSD* > 0.40 were discarded from the remaining set.

#### steric clash detection

After modeling the complete structure, models with steric clashes were discarded considering the C_*α*_ distance between loop residues and other residues of the protein, using a cut-off value of 3*Å*.

### Model building

Model generation was done using a two stage procedure. First the candidate loops were superimposed on the query flanks of the template, then MODELLER was used to generate a model of the un-gapped structure with the correct amino acid sequence.

### Model selection

To rank the models, we considered two scores. The first one is the *JSD* score (see above) and the second one is the Discrete Optimized Protein Energy (*DOPE*) score implemented in MODELLER^59^. *DOPE* is an atomic-distance-dependent statistical potential derived from known protein structures. Our procedure returns a maximum of 10 models per loop, corresponding to the 5 models with the lowest *JSD* score, and 5 models with the lowest *DOPE* score. It has to be mentioned that some degrees of overlap may occur among the top 5 models selected by each score. This may lead to smaller number of final models (<10 models).

### Loop quality assessment

To assess the quality of the results, we use the RMSD of the loop candidates main chain heavy atoms (N, C_*α*_, C′ and O). Consistently with previous studies^32,36,43^, we use different RMSD values. The local RMSD corresponds to the RMSD measured after performing the best fit superimposition of the loop region only. In the flanked RMSD, the flanks are first superimposed, excluding the loop atoms, and the RMSD is calculated over the loop region. In the global RMSD, the template structure is superimposed on the target structure excluding the loop region, then the RMSD is calculated over the loop of interest.

### Solvent accessibility of the loops

We measured the solvent accessibility of the loop residues using Naccess^60^. Residues with relative solvent accessibility (RSA) ≤ 20% were considered as buried. Defining a loop as buried if less than 25% of its residues are exposed, no loop in the three test sets is buried. The median percentage of buried residues are of 29, 33 and 17% for the CASP11, CASP12 and HOMSTRAD sets, respectively.

### Comparison with other approaches

In this work we compare the performance of our loop modeling protocol with two state-of-the-art *ab initio* methods - GalaxyLoop-PS2^32^ and Rosetta Next-generation KIC (NGK)^31^, one state-of-the-art data-based approach - LoopIng^37^ and one hybrid method - Sphinx^41^. The NGK runs were performed using the protocol provided by^31^, using Rosetta energy values to rank the models. GalaxyWEB was used to generate the GalaxyLoop-PS2 results. Since GalaxyWEB returns only 5 models, and does not return scores, we repeated the GalaxyWEB protocol two times to obtain 10 models per loop. Furthermore, GalaxyWEB does not accept loop modeling for loops of size more than 20 amino acids or loops belonging to proteins of more than 500 residues, which made the comparison impossible for 43 loops over the total of 168 (26% of the cases). LoopIng results were obtained using the LoopIng web-server. It can generate 10 models per loop, and returns only the loop regions, supplemented by two residues on each side of the loop. Since we use flanks of 4 amino acids, and to compare our results in a fair manner, we considered a flank size of 2 amino acids for the comparison with LoopIng. Furthermore, the web-server accepts loops of size 4 to 23 amino acids. Consequently, the comparison is not possible for 14 loops over the total of 168 (8% of the cases). We used Sphinx web-server to obtain loop predictions for all the loops in our test sets. Table 3 summarizes the number of loops considered for performance comparisons. We distinguish between *ab initio* and data-based search methods. Loop subsets that could be predicted by groups of approaches (Common subsets) are identified.

**Table 3:** Loop number for CASP11, CASP12 and HOMSTRAD test sets.

Size	CASP11	CASP12	HOMSTRAD	all
	69 (21)	76 (18)	23	168 (39)
DaReUS-Loop	67 (20)	75 (17)	23	165 (37)
NGK	66 (18)	76 (18)	23	165 (36)
GalaxyLoop-PS2	50 (9)	56 (9)	19	125 (18)
**Common**_*ai*_	47	55	19	121
LoopIng	63 (15)	69 (13)	22	154 (28)
Sphinx	69 (21)	76 (18)	23	168 (39)
**Common**_*db*_	62	69	22	153
*min*(*JSD*) ≤ 0.2	53 (8)	59 (6)	23	135 (14)
NGK	51 (6)	59 (6)	23	133 (12)
GalaxyLoop-PS2	40 (1)	46 (4)	19	105 (5)
**CommonHC**_*ai*_	40	46	19	105
LoopIng	51 (6)	55 (4)	22	128 (10)
Sphinx	53 (8)	59 (6)	23	135 (14)
**CommonHC**_*db*_	51	55	22	128

size: number of loops identified. DaReUS-Loop, NGK, GalaxyLoop-PS2, LoopIng and Sphinx: number of loops that could be modeled using each approach. The number of long loops (at least 15 residues) are reported within parentheses. Common: number of target loops predictable by all different approaches, distinguishing ab initio (_ai) and data-based approaches (_db). CommonHC: subset of Common corresponding to loops predicted with a high confidence index (JSD *leq* 0.2, see below).

## Electronic supplementary material

Supplementary information

## Data Availability

The set of all gapped models for CASP11 and CASP12 generated and analysed during the current study are available with the sequence of the targets at http://bioserv.rpbs.univ-paris-diderot.fr/public/DaReUS-Loop.tgz. It contains, the top 10 predictions of every method (DaReUS-Loop, Rosetta NGK, GalaxyLoop-PS2, LoopIng and Sphinx) and the corresponding RMSD values. It also includes a script that can be used to measure the RMSD values, as well as a detailed description (README.txt) on the data and how to use the script.
