# Supplementary material for: DaReUS-Loop: accurate loop modeling using fragments from remote or unrelated proteins
Source: Sci Rep. 2018 Sep 12;8:13673. doi: 10.1038/s41598-018-32079-w (PMC6135855; doi:10.1038/s41598-018-32079-w)
Supplement: Supplementary file 1 — Supplementary information [file 41598_2018_32079_MOESM1_ESM.pdf]

DaReUS-Loop: accurate loop modeling using fragments from remote or unrelated proteins

Yasaman Karami[1], Frédéric Guyon[1], Sjoerd De Vries[1,*], and Pierre Tufféry[1,*]

[1]Molécules Thérapeutiques in silico, UMR-S973, Institut National de la Santé et de la Recherche Médicale (INSERM), Université Paris Diderot, Sorbonne Paris Cité, RPBS, 75013 Paris, France.
[*]sjoerd.de-vries@inserm.fr, pierre.tuffery@univ-paris-diderot.fr

ABSTRACT

Despite efforts during the past decades, loop modeling remains a difficult part of protein structure modeling. Several approaches have been developed in the framework of crystal structures. However, for homology models, the modeling of loops is still far from being solved. We propose DaReUS-Loop, a data-based approach that identifies loop candidates mining the complete set of experimental structures available in the Protein Data Bank. Candidate filtering relies on local conformation profile-profile comparison, together with physico-chemical scoring. Applied to three different template-based test sets, DaReUS-Loop shows significant increase in the number of high-accuracy loops, and significant enhancement for modeling long loops. A special advantage is that our method proposes a prediction confidence score that correlates well with the expected accuracy of the loops. Strikingly, over 50% of successful loop models are derived from unrelated proteins, indicating that fragments under similar constraints tend to adopt similar structure, beyond mere homology.

Table S1. Prediction results over the top 2 models. The average and its standard deviation and median RMSD values reported in Å. The RMSDs are calculated as root-mean-square deviation of the best candidate loop main-chain atoms N, C_α, C and O to the native loop. Bold values correspond to the best values among all the methods.

subset	method	CASP11	CASP12	HOMSTRAD	$< 1Å$ (%)	$< 2Å$ (%)
Common_{ai}	best	2.18	2.31	1.65	24	66
	DaReUS-Loop	**3.34**	**3.63**	**2.80**	**12**	**34**
	Rosetta-NGk	3.48	4.17	3.05	8	28
Common_{db}	DaReUS-Loop	**3.52**	**3.94**	2.98	**10**	**33**
	Sphinx	4.58	4.47	**2.88**	9	30
CommonHC_{ai}	best	1.43	1.63	1.65	28	76
	DaReUS-Loop	**2.45**	**2.84**	**2.80**	**14**	**39**
	Rosetta-NGk	3.01	3.67	3.05	9	31
CommonHC_{db}	DaReUS-Loop	**2.56**	**2.81**	2.98	**12**	**39**
	Sphinx	3.58	3.65	**2.88**	11	36

Table S2. Modeling long loops. The flanked RMSDs of all the long loops (at least 15 residues) are reported for all the methods. The RMSDs are calculated as root-mean-square deviation of the loop main-chain atoms N, C_{α}, C and O to the native loop, after superimposition of the flanks. [a] Outliers.

test-set	target	loop name	Loop size	DaReUS -Loop	Rosetta-NGK	GalaxyLoop -PS2	DaReUS -Loop	LoopIng	Sphinx
CASP11	T0807	A16-Q30	15	1.26	3.05	2.87	0.65	7.77	0.87
CASP11	T0817	G350-L364	15	8.99	5.57		8.81	8.67	5.73
CASP12	T0928	A203-K218	16	1.99	0.89	1.43	2.00	9.26	1.31
CASP12	T0928	L352-T367	16	3.46	4.21	5.64	3.24	6.54	4.53
CASP11	T0800	N68-E83	16				6.28	4.71	5.98
CASP12	T0902	S224-K240	17	5.23	6.69	3.13	5.52	6.22	6.05
CASP12	T0909	G183-Q199	17	2.24	18.34[a]	18.15[a]	2.03	10.61	20.17[a]
CASP11	T0817	F65-L81	17	1.27	4.79		1.24	8.49	5.32
CASP11	T0766	H75-S91	17				1.59	5.97	3.34
CASP11	T0854	S130-P151	22	1.21	3.53		1.25	7.62	4.48
CASP12	T0920	G317-Y339	23	4.39	7.49				
CASP12	T0928	I269-C280	23				4.94		13.74
CASP11	T0760	L55-Q78	24	3.60	11.30		3.65		0.51
CASP12	T0889	G184-E210	27	6.15	7.98		5.56		9.95
CASP11	T0817	H311-D338	28	4.68	6.68		6.35		26.91[a]

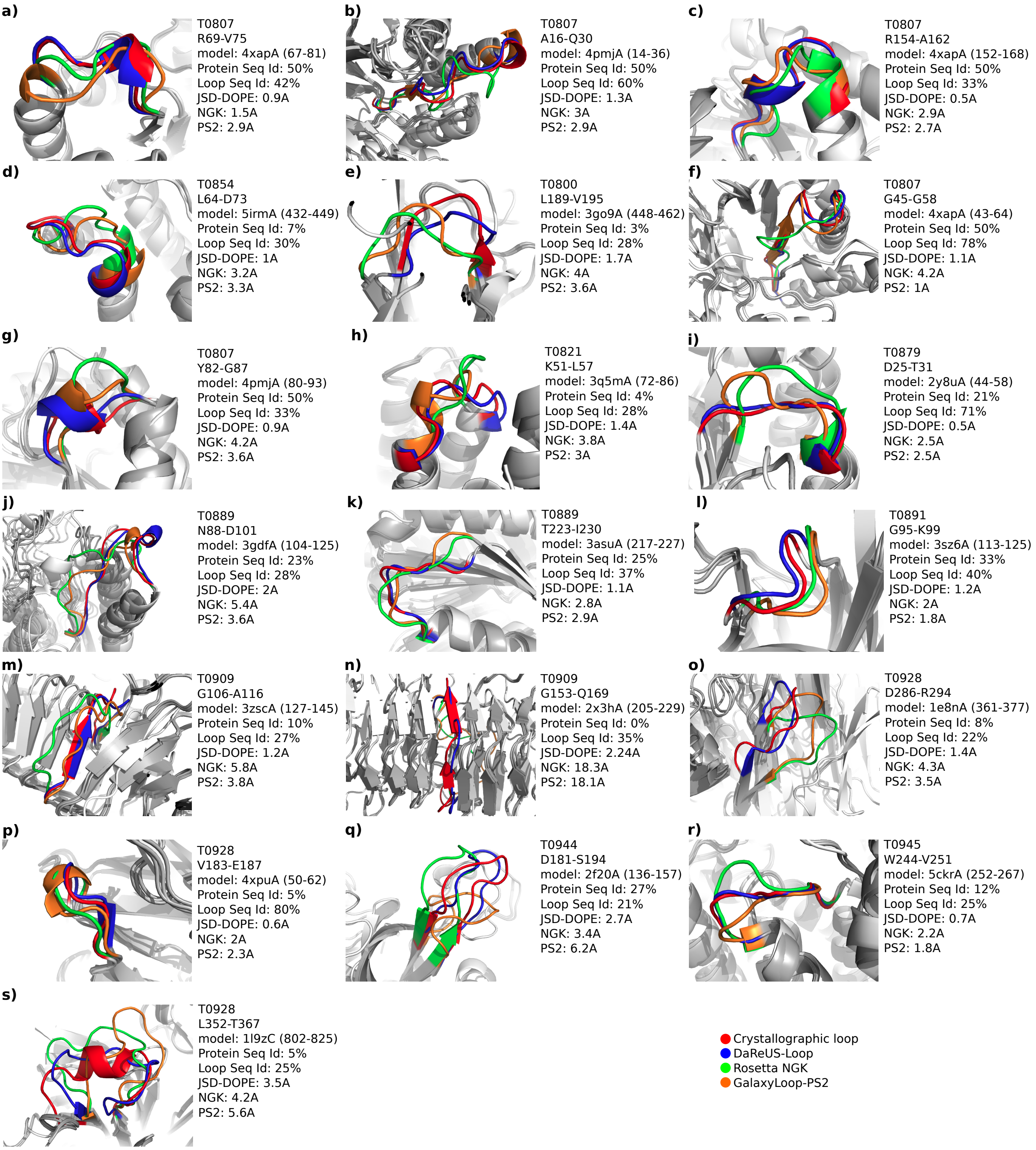

Figure S1. Examples of loop modeling in CASP test sets. Illustrative examples of predicted loops are shown (a-s) to compare DaReUS-Loop (blue) with GalaxyLoop-PS2 (orange) and Rosetta NGK (green). The crystal structure of the loops is colored in red. The CASP targets and loop residues are reported. In addition, the percentage of sequence identity between the best candidates selected by DaReUS-Loop and targets are shown, over both the loops and entire protein sequences. In addition, the flanked RMSD values are reported for the predictions of every model.
